# A comparative study of grain quality and physicochemical properties of premium japonica rice from three typical production regions

**DOI:** 10.3389/fpls.2024.1270388

**Published:** 2024-01-25

**Authors:** Zhi Dou, Qian Yang, Halun Guo, Yicheng Zhou, Qiang Xu, Hui Gao

**Affiliations:** ^1^ Jiangsu Co-Innovation Center for Modern Production Technology of Grain Crops, Yangzhou University, Yangzhou, China; ^2^ Jiangsu Provincial Key Laboratory of Crop Genetics and Physiology, Yangzhou University, Yangzhou, China; ^3^ College of Agriculture, Yangzhou University, Yangzhou, China

**Keywords:** japonica rice, three production regions, grain quality, physicochemical properties, starch fine structure, relation

## Abstract

Grain quality indicates rice commodity value. This research compared grain quality and physicochemical properties of premium japonica rice from three production regions, Yangtze River downstream of China (YRDCN), Northeast region of China (NECN) and Japan. Results showed that there were distinct quality and physicochemical characteristics variance among the three groups of japonica rice, while CVs of most quality parameters from low to high was Japan, YRDCN and NECN. YRDCN rice presented obvious lower apparent amylose content (AAC) and ratio of each chain-length sections of amylopectin, and showed higher protein contents especially glutelin and ratio in short and intermediate amylopectin molecules. Among three rice groups, YRDCN rice presented weaker appearance, whereas did not show inferior cooking and eating properties, which was primarily linked to lower AAC. Rice AAC and starch fine structure significantly correlated with pasting parameters, swelling power and solubility, while protein content had close relation with taste analyzer parameters. Results of this study indicated improvement direction for japonica rice of YRDCN, and also provided reference for consumers’ rice purchasing selection in accordance with individual taste preference.

## 1 Introduction

Rice is the staple food for over half of the global population, and approximately 90% of the global rice is produced and consumed in Asia (Bandumula, 2018). In countries where rice is consumed, excellent-quality rice is more acceptable by consumers and shows higher commodity value. Rice is divided into two sub-species, indica rice and japonica rice. In East Asia, most residents prefer japonica rice, as it is more pliable and has an elastic mouthfeel than indica rice. Japan carried out an excellent-eating quality japonica rice breeding program in the 1950s and successfully bred a number of famous japonica rice varieties, such as Koshihikari, a classical premium rice variety that had been introduced for the cultivation of high-grade commodity rice by China, Australia, America and other countries (Kobayashi et al., 2018). So far, Japan’s japonica rice varieties are more world-renowned than japonica rice from other countries and have more expensive market prices. China is the largest japonica rice-producing and rice-consuming country in the world. The northeast region, its largest-scale japonica rice planting region, also leads in excellent-quality japonica rice breeding in China. Wuyoudao4, the most representative rice variety of this region, had gained great public praise and also usually presented higher prices than other varieties in China. The above-mentioned two japonica rice-producing regions have lower grain-filling temperatures and larger diurnal temperature ranges, which are favorable for excellent-quality formation. Yangtze River downstream of China (YRDCN) is the second-largest japonica rice-producing region in China, and the grain quality of japonica rice from this region was universally considered inferior to those from the northeast region of China (NECN) and Japanese rice in consumers’ impression. YRDCN rice production usually encounters higher grain-filling temperatures, which would deteriorate grain quality. Moreover, farmers in YRDCN used to apply larger quantities of nitrogen fertilizer to obtain high yields. In recent years, rice breeders from the Yangtze River downstream of China bred a number of so-called semi-glutinous (apparent amylose content<13%) rice varieties with good taste, in accordance with the local consumers’ preference for soft and sticky rice taste (Wang et al., 2022). However, there have been few studies concerning grain quality differences of current premium japonica rice from the three typical rice production regions.

Rice quality is defined by its appearance, cooking properties, sensory taste, and nutrition. Rice appearance quality is the first criterion, directly affecting consumers’ purchasing desire (Custodio et al., 2019); it mainly includes grain shape, transparency, and chalk characteristics. Cooking and eating qualities, the most vital quality characteristics for rice consumers, principally contain rice starch solubility and swelling ability in hot water and hardness, stickiness, elasticity, and aroma of cooked rice. Eating properties can be estimated by instruments or panelists. No doubt, the panelists’ points system is the “most real standard” in food sensory evaluation, but it requires abundant tested samples, complex test procedures, and enough experienced panelists. Instrument measurements were universally used to assess rice cooking and eating quality parameters, such as rapid visco analyzer (RVA), rice taste analyzer (RTA), and texture profile analysis (TPA) due to their high efficiency, repeatability, and objectivity (Li et al., 2016; Sultana et al., 2022; Zhu et al., 2022). Rice nutrition contains protein and mineral elements, but they are mostly stored in bran layers, whereas rice is mainly consumed as white polished grains due to its more favorable appearance, palatability, and ease of cooking, compared with brown rice. Hence, milled rice nutrition is less mentioned in premium quality assessment, unless in food-deficient regions.

Starch, the primary substance in rice endosperm, is a branched glucose polymer comprising two categories of molecules: amylose and amylopectin. Amylose has a relatively small molecular weight (~10^5–6^) with a few long branches and accounts for 0%–30% of total rice starch, while amylopectin has a higher molecular weight (~10^7–8^) with a mass of short branches and accounts for 70% to 100% of total rice starch (Amagliani et al., 2016). Amylose content has been considered a crucial predictor of rice cooking and eating quality. Rice grains with lower amylose content tend to have a softer mouthfeel and present a longer time to retrogradation of cooked rice. In addition to amylose content, starch’s fine structure also plays a critical role in grain physicochemical properties formation. It is actually the complexity results from various forms, majorly including the relative proportion of amylose and amylopectin molecules, and amylopectin chain-length distribution and associated degree of branching (Patindol et al., 2015). The diversity of starch components and fine structure across rice germplasms significantly determines different rice physicochemical properties, thereby resulting in distinctive cooking and eating quality for specific rice varieties (Noda et al., 2003; Li et al., 2016; Cao et al., 2018; Wang et al., 2020).

Protein, the second most abundant substance of rice endosperms after starch, could be divided into albumins, globulins, prolamins, and glutelins, depending on their solubility characteristics (Kubota et al., 2010). Protein content and its components also show considerable influence on rice quality parameters (Martin and Fitzgerald, 2002; Baxter et al., 2014; Balindong et al., 2018). Protein in rice endosperm basically exists around amyloplast in the form of discrete particles called protein bodies. Higher protein content tends to make rice taste harder and less sticky since too many protein bodies would limit starch dissolved in water during the cooking process (Li et al., 2022).

This research compared grain quality and physicochemical properties of excellent-quality japonica rice from the Yangtze River downstream and northeast region of China and Japan; the relationship between rice grain components, starch fine structure, and physicochemical properties was also explored. The main objectives of this study were to determine the rice quality difference of premium rice from three typical japonica rice regions and preliminarily the physicochemical mechanism behind the grain quality differences. Results would indicate the direction of japonica rice quality improvement in the Yangtze River downstream, China, and also provide selection reference for rice consumers in accordance with individual taste preferences.

## 2 Materials and methods

### 2.1 Materials

Eighteen japonica rice varieties samples were collected, including six varieties from the Yangtze River downstream of China, six varieties from the northeast region of China, and six varieties from Japan; all varieties were released in the recent 15 years except for Koshihikari, a constant popular variety for more than 60 years in Japan. Each tested rice material was acknowledged as an excellent-quality variety at least in rice-producing regions. Rice grains of each variety were produced from respective suitable planting regions in 2019. The detailed information on each rice variety sample is displayed in **Table 1**.

**Table 1:** Origin of tested rice samples.

Variety	Abbreviation code	Sample collection	Country	Detailed origin region	Released year
Nangeng46	NG46	Produced us	China	Jiangsu province, Yangtze River downstream	2008
Suxianggeng100	SXG100	Produced us	China	Jiangsu province, Yangtze River downstream	2015
Nangeng9108	NG9108	Produced us	China	Jiangsu province, Yangtze River downstream	2013
Xudao9	XD9	Produced us	China	Jiangsu province, Yangtze River downstream	2015
Songxianggeng1018	SXG1018	Offered by local research institution	China	Shanghai City, Yangtze River downstream	2017
Huruan1212	HR1212	Offered by local research institution	China	Shanghai City, Yangtze River downstream	2017
Wuyoudao4	WYD4	Offered by local research institution	China	Heilongjiang province, Northeast region	2009
Jigeng816	JG816	Offered by local research institution	China	Jilin province, Northeast region	2018
Jigeng515	JG515	Offered by local research institution	China	Jilin province, Northeast region	2016
Jinongda667	JND667	Offered by local research institution	China	Jilin province, Northeast region	2019
Longdao18	LD18	Offered by local research institution	China	Heilongjiang province, Northeast region	2014
Songgeng22	SG22	Offered by local research institution	China	Heilongjiang province, Northeast region	2016
Koshihikari	Kos	Bought from supermarket	Japan	Niigata Prefecture	1953
Shinnosuke	Shi	Bought from supermarket	Japan	Niigata Prefecture	2015
Tsuyahime	Tsu	Bought from supermarket	Japan	Yamagata Prefecture	2010
Yukiwakamaru	Yuk	Bought from supermarket	Japan	Yamagata Prefecture	2015
Seitennohekireki	Sei	Bought from supermarket	Japan	Aomori Prefecture	2015
Yumepirika	Yum	Bought from supermarket	Japan	Hokkaido	2009

### 2.2 Measuring methods

#### 2.2.1 Grain shape and transparency

For each variety, 30 grains were randomly selected for the determination of grain length, width, and thickness using a digital caliper (DL92150P, Deli, Zhejiang, China). Grain transparency was evaluated using a rice grain scanner (Mrs-9600TFU2L, Zhongjing, Shanghai, China). Grain transparency phenotypes of two varieties each from YRDCN, NECN, and Japan were photographed using a stereomicroscope (S8 APO, Leica, Wetzlar, Germany).

#### 2.2.2 Amylose content

The amylose content of rice grains was determined according to the method reported by Juliano et al. (1981).

#### 2.2.3 Contents of four protein components

Protein fractions were separated and measured based on the method of Liu et al. (2005). The general process involved sequential extraction of rice powders, as follows: water, generating albumins; 10% NaCl, generating globulins; 55% *n*-propanol, generating prolamins; and Biuret reagent, generating glutelins. Glutelin content was tested using the Biuret method, while the contents of the other three fractions were conducted using the Bradford reagent.

#### 2.2.4 Starch extraction of rice grains

Starch extraction from rice grains was undertaken based on the method reported elsewhere (Wei et al., 2010; Wang et al., 2020).

#### 2.2.5 Molecular size of whole branched starch molecules

The structure of extracted whole starch molecules was characterized using an LC20-AD system (Shimadzu Corporation, Kyoto, Japan) connected with a differential refractive index (DRI; Shimadzu). A combination of GRAM pre-column, GRAM 30, and GRAM 3000 analytical columns (PSS, Mainz, Germany) was used to acquire the size distribution of whole starch molecules. Chain-length distributions (CLDs) were obtained using a combination of GRAM pre-column, GRAM 100, and GRAM 1000 analytical columns (PSS). The calculation methods were based on the information reported by Tao et al. (2019) and Wu et al. (2014). Amylopectin was divided into three chain-length sections according to recent literature (Li and Liu, 2019), and amylose was divided into three chain-length sections as described in the study of Li et al. (2016).

#### 2.2.6 Relative crystallinity

Rice starch relative crystallinity was measured using an X-ray powder diffractometer (D8 Advance, Bruker, Karlsruhe, Germany) and calculated using the MDI Jade 6 software.

#### 2.2.7 RVA parameters

Pasting properties of rice flour were assayed using a Rapid Viscosity Analyzer (RVA-Tech Master, Newport Scientific Pty. Ltd., Warriewood, NSW, Australia).

#### 2.2.8 Solubility and swelling power

Rice starch solubility and swelling power were determined and calculated using the method reported by Hasjim et al. (2012).

Starch samples (m0) were mixed with water (2%, w/v) and placed in a 2-mL centrifuge (m1) for a bath at 95°C for 30 min. After cooling at ambient temperature, the solutions were centrifuged at 4,000 *g* for 12 min. Then, the liquid supernatants were discarded. The colloid remaining in the centrifuge tube was weighed (m2), and the sediments were dried to a constant weight (m3) at 60°C. The solubility and swelling power (SP, g/g) were calculated according to the following equations:


Solubility (%)=100×(m0+m1−m3)/m0×100%

Swelling power (g/g)=(m2−m1)/(m3−m1)

#### 2.2.9 Taste value parameters

The taste value was assayed using a taste analyzer (STA1A, SATAKE, Tokyo, Japan), and Japanese japonica rice was used as a detection line, in accordance with the detailed procedure described in Zhu et al. (2022). A sample of 30 g of polished grains was placed into a stainless steel tank, and water was added to form the ratio of rice to water to 1:1.33. The sample was then soaked for 30 min, covered with filter paper, and sealed with a rubber ring. The stainless steel tank was steamed in the electric rice cooker for 30 min and kept warm for 10 min; then, it was taken out from the rice cooker, and the steamed rice was gently stirred and turned over. Then, the tank was covered with filter paper and put into the supporting air cooling device for cooling for 20 min. After cooling, the filter paper was replaced by the supporting steel cover, and the steamed rice was sealed and cooled for 90 min; 8 g of steamed rice was placed into a stainless steel ring and then crushed into a cake to be measured. The rice cake was placed in the measuring tank, and the rice taste analyzer was used to assay the sample’s hardness, stickiness, and taste value. Three rice cakes were assayed for every sample.

#### 2.2.10 Statistical analysis

Excel 2013 was used to calculate the mean value, standard error, and variable coefficient. One-way analysis and correlation analysis were conducted using Statistical Package SPSS 25.0. Significance was considered at *p*< 0.05.

## 3 Results

### 3.1 Grain appearance

Grain transparency and shape were measured to reflect rice appearance quality. In this experiment, the lower the transparency value, the higher the grain transparency. The transparency values of all YRDCN rice varieties were equal to or greater than 3, while transparency values of rice varieties of NECN and Japan were equal to or lower than 2 (**Table 2**), indicating that YRDCN rice was distinctly less transparent than NECN and Japanese rice.

**Table 2:** Comparison of japonica rice grain appearance quality among three production regions.

Source	Variety	Transparency	Length (mm)	Width (mm)	LWR	Thickness (mm)
YRDCN	NG46	5.0	4.831	2.920	1.656	2.194
	SXG100	3.3	4.697	2.890	1.629	2.231
	NG9108	4.7	4.680	2.896	1.619	2.198
	XD9	3.0	4.921	2.726	1.808	2.040
	SXG1018	3.7	5.109	2.909	1.759	2.198
	HR1212	3.3	4.896	2.639	1.858	2.023
	Mean	3.9a	4.856a	2.830a	1.722a	2.147a
	CV	21.49	3.28	4.17	5.86	4.23
NECN	WYD4	2.0	6.046	2.396	2.525	1.964
	JG816	1.7	4.477	2.761	1.625	2.082
	JG515	2.0	4.373	2.639	1.665	2.065
	JND667	2.0	4.338	2.688	1.615	2.027
	LD18	1.0	5.811	2.465	2.361	1.784
	SG22	1.0	6.276	2.374	2.647	1.861
	Mean	1.6b	5.220a	2.554b	2.073a	1.964b
	CV	16.23	17.55	6.39	23.48	6.07
Japan	KOS	1.7	4.718	2.758	1.713	2.031
	SHI	2.0	4.899	2.844	1.725	2.009
	TSU	1.7	4.858	2.768	1.758	2.015
	YUK	2.0	4.871	2.778	1.755	2.041
	SEI	2.0	4.957	2.717	1.826	2.011
	YUM	2.0	4.730	2.693	1.759	2.000
	Mean	1.9b	4.839a	2.760a	1.756a	2.018b
	CV	8.15	1.97	1.90	2.24	0.75

Values labeled by the different letters are significantly different (p< 0.05).

LWR, length–width ratio; Mean, mean value; CV, coefficient of variation.

Six Japanese rice varieties presented clearly much lower coefficients of variation (CVs) in grain shape parameters than the other two types of rice. Grain length, width, and length–width ratio of YRDCN ranged from 4.680 mm to 5.109 mm, 2.639 mm to 2.920 mm, and 1.619 mm to 2.231 mm, respectively, being close to Japanese rice generally, while YRDCN rice grains were thicker than Japan and NECN rice. CVs of NECN rice in grain length, width, length–width ratio (LWR), and thickness were much higher than those of the other two groups. The grain length of NECN rice ranged from 4.338 mm to 6.276 mm and exhibited significantly lower width than the other two groups; thus, we speculated that NECN rice varieties varied more in genetic background than the other two groups, as grain shape is more affected by genetic factor rather than cultivation methods and growth environment (Zhao et al., 2022). LD18 and SG22, two varieties from NECN, had much lower thickness than the remaining 16 varieties.

### 3.2 Rice grain components and starch fine structure

#### 3.2.1 AAC and AM–TS ratio

As presented in **Table 3**, apparent amylose content (AAC) varied much for rice from different production regions. All rice varieties from YRDCN showed much lower AAC than the other two groups of rice, ranging from 10.2% to 12.3% with a smaller CV, while varieties from NECN generally presented the highest AAC among three production regions, ranging from 16.6% to 22.0%, and its CV was also higher than that of the other two groups. Japanese rice had intermediate amylose contents and CVs among the three groups of rice, with AAC ranging from 14.2% to 16.8%. YUM had the lowest AAC among Japanese rice varieties. The specific value of amylose to total starch content (AM–TS ratio) was determined using the size exclusion chromatography (SEC) method in this study. Mean values of the AM–TS ratio from low to high were ordered in the following sequence: YRDCN, NECN, and Japan. Significant differences were detected between the two groups.

**Table 3:** Comparison of japonica rice amylose content, protein components, and relative crystallinity among three production regions.

Source	Variety	AAC (%)	AM–TS ratio (%)	CP (%)	Albumin (mg/g)	Globulin (mg/g)	Prolamin (mg/g)	Glutelin (mg/g)	TP (mg/g)	RC (%)
YRDCN	NG46	12.33	11.50	6.72	3.02	3.91	6.80	37.08	50.80	19.66
	SXG100	11.33	7.62	6.88	3.48	3.41	6.35	39.26	52.50	20.15
	NG9108	11.90	8.39	7.78	5.17	5.04	6.86	47.55	64.62	20.32
	XD9	12.72	9.35	7.71	4.40	4.71	6.77	48.80	64.67	19.98
	SXG1018	10.03	7.82	7.55	5.05	3.91	6.80	38.30	54.05	19.65
	HR1212	12.25	8.26	6.48	3.68	3.67	6.10	36.54	49.98	20.09
	Mean	11.77c	8.83c	7.19a	4.13b	4.11a	6.61a	41.25a	56.10a	19.98a
	CV	8.25	16.35	7.80	21.25	15.33	4.73	13.23	12.06	1.36
NECN	WYD4	19.30	18.85	6.00	4.08	4.04	6.68	27.75	42.55	15.55
	JG816	16.34	16.68	6.47	2.86	3.28	5.95	36.98	49.06	17.41
	JG515	17.85	18.88	6.13	4.10	4.21	6.63	31.56	46.49	15.40
	JND667	17.17	16.52	7.17	3.15	3.25	6.24	42.46	55.10	17.62
	LD18	21.98	20.42	7.53	2.88	4.04	6.92	40.43	54.27	16.47
	SG22	20.48	19.94	6.79	3.90	4.27	7.48	42.35	58.00	15.88
	Mean	18.86a	18.55a	6.68ab	3.49b	3.85a	6.65a	36.92ab	50.91a	16.39c
	CV	11.32	8.77	8.97	17.09	12.03	8.03	16.49	11.52	5.80
Japan	KOS	17.50	15.57	5.81	5.43	3.66	5.96	34.06	49.12	17.23
	SHI	15.47	16.29	5.93	5.89	3.57	6.27	33.00	48.72	18.01
	TSU	14.89	16.76	6.25	5.51	4.01	6.16	34.55	50.23	17.25
	YUK	15.31	15.87	5.81	5.02	3.73	5.95	31.54	46.24	17.57
	SEI	17.93	15.99	6.17	5.71	4.08	6.38	31.77	47.93	17.70
	YUM	14.18	13.06	7.06	5.47	4.42	7.13	38.93	55.95	16.44
	Mean	15.88b	15.62b	6.17b	5.51a	3.91a	6.31a	33.97b	49.70a	17.37b
	CV	9.42	8.36	7.66	5.31	8.20	6.90	7.97	6.72	3.11

Values labeled by the different letters are significantly different (p< 0.05).

AAC, apparent amylose content; AM–TS ratio, amylose–total starch ratio; CP, crude protein; TP, total protein, the sum of the four components; RC, relative crystallinity; Mean, mean value; CV, coefficient of variation.

#### 3.2.2 Protein components

Crude protein content was determined using the Kjeldahl nitrogen method. The average crude protein content from high to low was ordered in the following sequence: YRDCN, NECN, Japan. Significant differences were observed between YRDCN and Japanese rice. Albumin, globulin, prolamin, and glutelin contents were determined in this study, and the sum of the four protein components (TPs) was also displayed, while TP value differences among the three rice groups were smaller than those of crude protein. Glutelin always occupied more than 70% of TP, and its average content in YRDCN rice was 11.4% higher than that of NECN rice and 21.4% higher than that of Japanese rice, which primarily resulted in the ranking of crude protein content and TP. The highest globulin content was also detected in rice from YRDCN, while Japanese rice varieties showed lower prolamin content than the two groups of rice from China. Though Japanese rice showed the lowest total protein contents among the three groups, its mean albumin content was 33.4% higher and 57.9% higher than YRDCN rice and NECN rice, respectively. Among Japanese rice varieties, YUM showed higher total protein and glutelin content than others.

#### 3.2.3 Starch fine structure

In addition to starch and protein components, the starch molecular structure also plays an important role in rice physicochemical properties, thereby affecting grain quality. The components with X< 100 are defined as amylopectin chains, while those with X ≥ 100 are defined as amylose chains (Vilaplana et al., 2012). The amylose CLDs have DPs ranging from 100 to 20,000, and amylose is further divided into three sections: 100 ≤ X< 1,000, 1,000 ≤ X< 2,000, and 2,000 ≤ X< 20,000. The proportion of each section was calculated (**Table 4**). Apparently, the three groups of japonica rice differed greatly in CLD features. For YRDCN rice, its proportion of amylopectin chains of 6 ≤ X< 32 was found to be significantly higher than that of the other two types of rice, NECN and Japanese rice exhibited a similar percentage of 6 ≤ X< 32, and NECN rice showed lower percentage in both the section of 32 ≤ X< 62 and 62 ≤ X< 100 than YRDCN rice and Japanese rice. The proportion of amylose CLD features of 100 ≤ X< 1,000, 1,000 ≤ X< 2,000, and 2,000 ≤ X< 20,000 all showed the following tendency: NECN rice > Japanese rice > YRDCN rice. The highest CVs were always detected in YRDCN rice.

**Table 4:** Comparison of japonica rice starch length-distribution parameters among three production regions.

Source	Variety	DP 6 ≤ X< 32 (%)	DP 32 ≤ X< 62 (%)	DP 62 ≤ X< 100 (%)	DP 100 ≤ X< 1,000 (%)	DP 1,000 ≤ X< 2,000 (%)	DP 2,000 ≤ X< 20,000 (%)
YRDCN	NG46	0.709	0.133	0.0190	0.0581	0.0204	0.0364
	SXG100	0.736	0.145	0.0201	0.0420	0.0132	0.0210
	NG9108	0.728	0.145	0.0202	0.0428	0.0154	0.0257
	XD9	0.723	0.145	0.0200	0.0487	0.0173	0.0276
	SXG1018	0.730	0.154	0.0218	0.0415	0.0138	0.0229
	HR1212	0.715	0.161	0.0252	0.0446	0.0149	0.0231
	Mean	0.723a	0.147a	0.0211ab	0.0463b	0.0158c	0.0261c
	CV	1.37	6.46	10.62	13.73	16.91	21.24
NECN	WYD4	0.657	0.123	0.0170	0.0861	0.0421	0.0602
	JG816	0.676	0.122	0.0152	0.0762	0.0375	0.0531
	JG515	0.654	0.128	0.0191	0.0887	0.0428	0.0573
	JND667	0.675	0.131	0.0172	0.0735	0.0374	0.0543
	LD18	0.642	0.128	0.0203	0.0994	0.0466	0.0581
	SG22	0.645	0.131	0.0208	0.0921	0.0447	0.0625
	Mean	0.658b	0.127b	0.0183b	0.0860a	0.0419a	0.0576a
	CV	2.21	2.96	11.74	11.36	8.95	6.16
Japan	KOS	0.661	0.153	0.0254	0.0783	0.0325	0.0449
	SHI	0.662	0.148	0.0240	0.0804	0.0344	0.0481
	TSU	0.660	0.146	0.0242	0.0822	0.0355	0.0498
	YUK	0.658	0.155	0.0260	0.0774	0.0332	0.0481
	SEI	0.671	0.143	0.0216	0.0773	0.0348	0.0478
	YUM	0.698	0.137	0.0188	0.0609	0.0284	0.0414
	Mean	0.668b	0.147a	0.0233a	0.0761a	0.0331b	0.0467b
	CV	2.30	4.47	11.56	10.11	7.81	6.51

Values labeled by the different letters are significantly different (p< 0.05).

Mean, mean value; CV, coefficient of variation.

### 3.3 Relative crystallinity

In rice starch granules, amylopectin, especially short-chain amylopectin, forms a crystalline region, whereas amylose mainly forms an amorphous region. The mean relative crystallinity from high to low has the following sequence: YRDCN, Japan, NECN. Significant differences were detected between any two groups, while the corresponding CV of the three groups of rice showed an oppositive sequence.

### 3.4 Rice cooking and eating parameters

#### 3.4.1 RVA parameters

The mean peak viscosity (PKV) from high to low has the following sequence: Japan, YRDCN, and NECN (**Table 5**). Rice samples from China varied largely within respective varieties, while a smaller variance was detected across Japan japonica rice samples, suggesting that Japan japonica rice displayed relatively stable RVA parameters among different varieties. The hot peak viscosity (HPV) of YRDCN and NECN was greatly lower than that of Japan and presented a higher CV. The mean cool paste viscosity (CPV) from high to low in turn has the following sequence, with obvious variance: Japan, NECN, and YRDCN. NG46, XD9, JG816, and JND667 showed marked higher CPV among China rice samples. A larger breakdown (BD) and related lower CV were found in rice samples of YRDCN than in NECN and Japan, illustrating that semi-glutinous japonica rice from YRDCN had better gel consistency. JND667 from NECN and Yku from Japan had approximate BD compared with the varieties from YRDCN. Compared with NECN rice and Japanese rice, YRDCN rice samples displayed clearly lower setback (SB), which is the recovery of the viscosity by cooling after heating starch suspension. This indicated that YRDCN rice could have better retrogradation performance upon cooling. YRDCN and Japan were generally similar in pasting temperature (PT) and were both obviously higher than those from NECN, reflecting the advantage of NECN rice in cooking than the other two groups.

**Table 5:** Comparison of japonica rice cooking and eating parameters among three production regions.

Source	Variety	Pasting parameters						PT (°C)	Taste analyzer parameters			Solubility (g/g)	Swelling power (%)
		PKV (cP)	HPV (cP)	BD (cP)	CPV (cP)	SB (cP)	Cos (cP)		Hardness	Stickiness	Taste value		
YRDCN	NG46	3,533.7	2,249.7	1,284.0	2,912.7	−621.0	663.0	71.7	5.60	8.76	83.60	11.87	10.93
	SXG100	2,833.7	1,379.3	1,454.3	1,920.3	−913.3	541.0	72.5	5.53	9.10	85.87	14.86	10.37
	NG9108	3,229.3	1,956.3	1,273.0	2,600.0	−629.3	643.7	73.6	6.10	8.23	78.03	12.35	11.00
	XD9	3,581.3	2,127.7	1,453.7	2,805.0	−776.3	677.3	73.6	6.31	7.14	72.66	12.99	10.18
	SXG1018	2,397.7	1,171.3	1,226.3	1,822.0	−575.7	650.7	72.3	5.96	7.03	74.73	13.87	12.46
	HR1212	2,862.3	1,623.7	1,238.7	2,264.0	−598.3	640.3	74.4	5.66	8.51	81.89	12.26	10.40
	Mean	3,073.0a	1,751.3b	1,321.7a	2,387.3c	−685.7b	636.0c	73.0a	5.86b	8.13a	79.46a	13.03a	10.89b
	CV	14.93	24.53	7.92	19.18	19.25	7.62	1.38	5.30	10.54	6.53	8.71	7.67
NECN	WYD4	2,536.3	1,832.3	704.0	2,910.3	374.0	1,078.0	69.9	6.02	8.62	80.18	6.10	13.16
	JG816	3,129.3	2,135.0	994.3	3,137.0	7.7	1,002.0	71.5	6.22	8.13	76.77	7.31	12.30
	JG515	2,617.3	1,759.0	858.3	2,801.0	183.7	1,042.0	69.6	6.39	8.16	76.10	9.41	13.16
	JND667	3,475.0	2,256.3	1,218.7	3,441.7	−33.3	1,185.3	72.0	6.77	7.48	70.94	11.35	12.74
	LD18	2,248.3	1,664.7	583.7	2,584.0	335.7	919.3	70.9	6.71	7.40	70.97	10.65	14.04
	SG22	2,350.0	1,751.0	599.0	2,743.7	393.7	992.7	71.0	6.70	7.29	70.46	9.81	15.18
	Mean	2,726.1a	1,899.7ab	826.3b	2,936.3b	210.2a	1,036.6b	70.8b	6.47a	7.85a	74.24a	9.11b	13.43a
	CV	17.28	12.55	30.07	10.51	89.57	8.71	1.30	4.75	6.79	5.42	22.24	7.73
Japan	KOS	3,184.3	2,139.3	1,045.0	3,269.0	84.7	1,129.7	74.7	6.60	7.83	73.27	14.19	15.95
	SHI	3,099.3	2,093.3	1,006.0	3,295.0	195.7	1,201.7	73.3	5.93	8.93	82.67	14.19	14.44
	TSU	3,264.0	2,352.0	912.0	3,534.7	270.7	1,182.7	73.6	5.70	9.03	84.53	16.73	13.85
	YUK	3,518.3	2,279.3	1,239.0	3,547.3	29.0	1,268.0	74.7	5.80	8.70	82.27	13.73	13.89
	SEI	3,129.7	2,344.3	785.3	3,653.7	524.0	1,309.3	74.4	5.83	9.00	83.63	11.71	14.28
	YUM	3,047.0	2,006.3	1,040.7	3,160.3	113.3	1,154.0	71.7	6.30	7.73	74.67	10.67	13.17
	Mean	3,207.1a	2,202.4a	1,004.7b	3,410.0a	202.9a	1,207.6a	73.7a	6.03b	8.54a	80.17a	13.54a	14.26a
	CV	5.29	6.51	15.06	5.70	88.17	5.69	1.54	5.78	7.00	6.1	15.71	6.56

Values labeled by the different letters are significantly different (p< 0.05).

PKV, peak viscosity; HPV, hot paste viscosity; BD, breakdown; CPV, cool paste viscosity; SB, setback; Cos, consistency; PT, pasting temperature; Mean, mean value; CV, coefficient of variation.

#### 3.4.2 Rice starch solubility and swelling power

The results of starch solubility and swelling power are displayed in **Table 5**. The solubility of NECN rice ranged from 6.10% to 11.35%, which was significantly lower than that of YRDCN and Japanese rice. Meanwhile, NECN rice simultaneously exhibited the highest starch solubility CV value among the three production regions. YRDCN rice presented significantly lower starch swelling power than the other two groups, suggesting that this type of rice requires less water for the cooking process than NECN and Japanese rice. The difference between NECN and Japanese rice was obviously smaller than the difference between YRDCN and NECN or Japanese rice. Starch swelling power CV values of the three types of rice were similar.

#### 3.4.3 Taste analyzer parameters

Rice hardness, stickiness, and taste value were measured using a rice taste analyzer. Taste values were generally similar between YRDCN and Japanese rice, and they both showed slight edges compared with NECN rice. There were eight varieties scoring more than 80 points, including four Japanese rice varieties (SHI, TSU, YUK, and SEI), three YRDCN rice varieties (NG46, SXG100, and HR1212), and one NECN rice variety (WYD4). In sum, grain hardness from low to high was ordered in the sequence YRDCN, Japan, and NECN rice. The grain hardness difference between NECN rice and the other two groups reached a significant level. Generally, Japanese rice varieties showed higher stickiness than the other two groups except for KOS and YUM, and it is notable that rice varieties have higher taste values accompanied by higher stickiness and lower hardness.

### 3.5 Correlation analysis of rice cooking and eating properties with grain components and starch fine structure

Correlation analysis indicated that relative crystallinity was significantly negatively related to amylose content and amylose ratio in DP 100 ≤ X< 1,000, 1,000 ≤ X< 2,000, and 2,000 ≤ X< 20,000, while it was significantly positively correlated with amylopectin ratio in 6 ≤ X< 32 and 32 ≤ X< 62.

BD was significantly negatively correlated with amylose content, AM–TS ratio, and DP 100 ≤ X< 1,000, 1,000 ≤ X< 2,000, and 2,000 ≤ X< 20,000. It was significantly negatively correlated with DP 6 ≤ X< 32 and 32 ≤ X< 62. SB and Cos were both significantly positively correlated with amylose content, AM–TS ratio, and proportion of DP 100 ≤ X< 1,000, 1,000 ≤ X< 2,000, and 2,000 ≤ X< 20,000, and they were significantly negatively correlated with DP 6 ≤ X< 32. The pasting temperature was significantly positively correlated with the ratio of DP 6 ≤ X< 32 and 32 ≤ X< 62 and positively correlated with DP 2,000 ≤ X< 20,000.

The proportions of amylose DP 100 ≤ X< 1,000, 1,000 ≤ X< 2,000, and 2,000 ≤ X< 20,000 were detected to be positively correlated with swelling power, while the proportion of amylopectin DP 6 ≤ X< 32 was negatively correlated with swelling power. Taste value and stickiness had a significant negative correlation with TP, glutelin, and crude protein (CP); stickiness was also significantly negatively correlated with prolamin. In addition, hardness was detected to be significantly positively correlated with AAC and AM–TS ratio and significantly negatively correlated with amylose DP 100 ≤ X< 1,000, 1,000 ≤ X< 2,000, and 2,000 ≤ X< 20,000.

## 4 Discussion

### 4.1 Differences in japonica rice grain appearance among the three production regions

**Figure 1:**
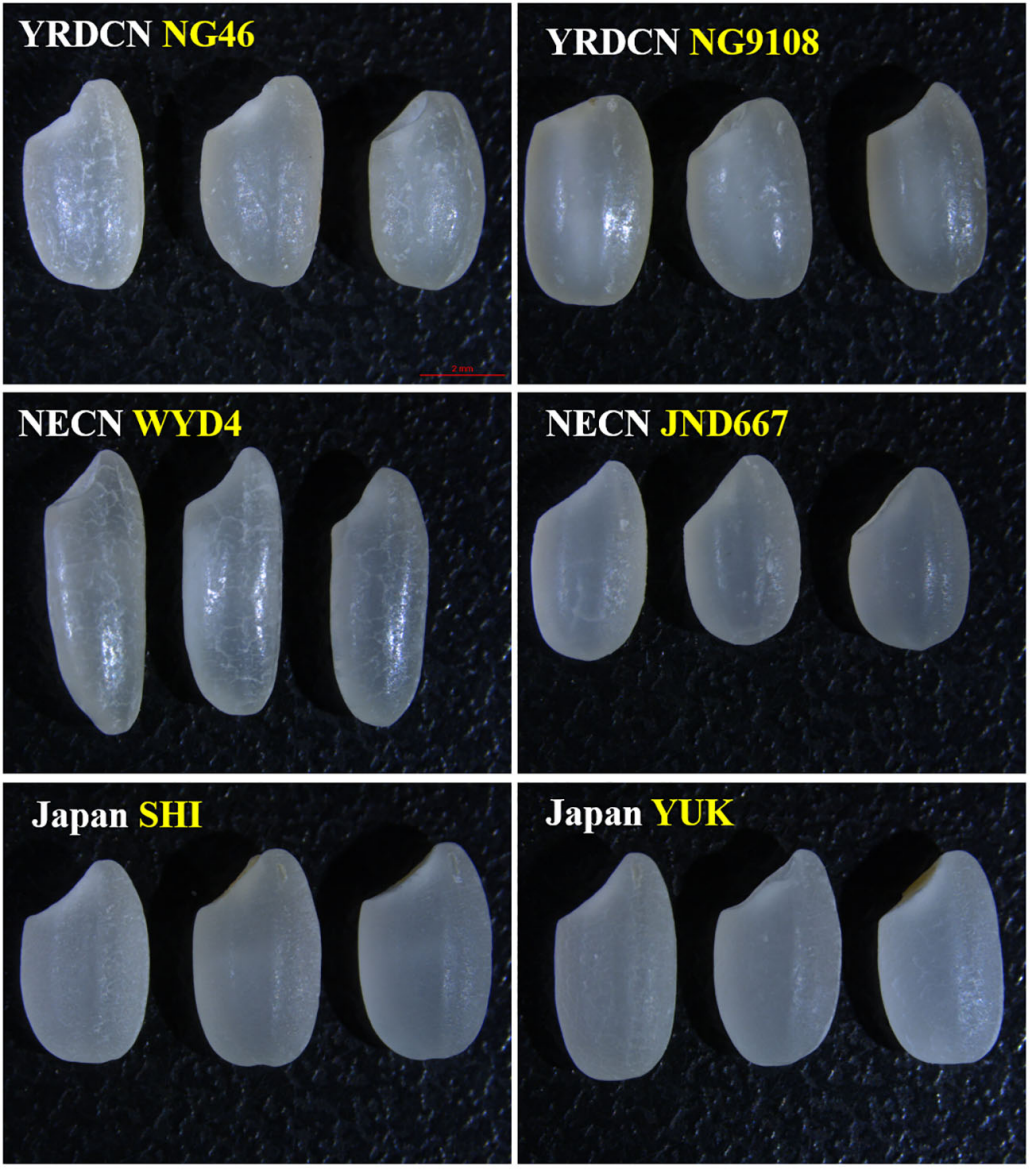
Comparison of transparency phenotype of some japonica rice varieties among three production regions. NG46, Nangeng46; NG9108, Nangeng9108; WYD4, Wuyoudao4; JND, Jinongda667; SHI, Shinnosuke; YUK, Yukiwakamaru; YRDCN, Yangtze River downstream of China; NECN, Northeast region of China.

Grain appearance majorly includes grain transparency and grain shape, and they significantly affect rice market prices and consumers’ acceptance. This research suggested that all YRDCN rice varieties were less transparent than NECN and Japanese rice. Other studies have also confirmed that the semi-glutinous japonica rice varieties from YRDCN usually presented dark or dull endosperm (Wang et al., 2017; Xu et al., 2020), which lowered their commercial quality. The visual transparency difference can be found in **Figure 1**. Lower amylose content and the resultant cavity in the center of starch granules have been confirmed to be responsible for the poor transparency of semi-glutinous rice varieties (Zhang et al., 2019). Six Japanese rice varieties generally had similar grain shapes, while YRDCN and NECN rice were not stable in grain shape among different varieties. Broadly speaking, uncooked rice grains with slender forms were found to be preferred in most consuming markets (Custodio et al., 2019). A comparison of grain shape showed that WYD4 and SG22 presented higher LWR than the other tested varieties, and they were indeed the more popular varieties due to both good-looking grain shape and favorable taste. Unfortunately, this research did not compare rice grain chalk characteristics, which was also considered an important rice appearance evaluation parameter, as some tested varieties were bought from supermarkets, which may be strictly screened during the process, whereas some were just processed with miniature instruments in our lab. The chalky rate of YRDCN rice was observed to be marked higher than that of NECN rice in previous studies (Yang et al., 2012; Zhang et al., 2021a).

### 4.2 Differences in japonica rice grain amylose and protein contents among the three production regions

Starch and protein occupy more than 95% substance in milled rice grains (Fitzgerald et al., 2009). Amylose content is broadly recognized as the significant parameter affecting rice cooking and eating quality; cooked rice with high amylose content shows a dry, hard, separate, less sticky mouthfeel and is visually dull and lacking gloss (Fitzgerald et al., 2009; Li et al., 2016; Shi et al., 2022). In this research, AACs in YRDCN rice ranged from 10.03% to 12.72%, being greatly lower in comparison with NECN rice (16.34%–21.98%) and Japanese rice (14.18%–17.93%). NECN rice varieties generally showed higher amylose contents and AM–TS ratios than Japanese rice, whereas part of the rice varieties in the two groups did not follow this rule. The characteristics of low amylose content in YRDCN japonica rice varieties were principally determined by the low-amylose-associated gene selection in breeding, such as *WXmp*, *WXmq*, and *WXmw* (Wang et al., 2017). Lower protein content had been the standing goal of japonica rice breeding and cultivation, especially in Japan. In rice endosperm, protein bodies exist around amyloplast, and they interact with amyloplast to form a network structure during rice gelatinization, thus restraining the swelling of starch, which would bring a hardness mouthfeel and increase the difficulty of rice cooking. Our research also testified the advantage of Japanese rice in maintaining lower protein content, but the protein difference between Chinese and Japanese rice varieties was smaller than the gap in a similar comparison conducted by another study using numerous common China japonica rice varieties (Nakamura et al., 2016), indicating that premium japonica rice breeding in China had made progress in protein content control, especially observed in YRDCN rice, which was liable to accumulate protein because of its marked higher grain-filling temperature than the other two regions. We noticed that the most renowned excellent-eating varieties all presented lower protein content in their respective production region, such as NG46 from YRDCN, WYD4 from NECN, and KOS and SHI from Japan. This discovery indirectly stated the crucial role of lower protein content in excellent eating quality formation. We thought that the higher grain protein content in YRDCN rice was linked to the higher nitrogen fertilizer application rates (Guo et al., 2017) and grain-filling temperature (Dou et al., 2018) in local rice production.

### 4.3 Differences in japonica rice cooking and eating quality among the three production regions and their relation with rice components and starch fine structure

Relative crystallinity had a negative correlation with amylose content and proportions of each amylose chain-length section (100 ≤ X< 1,000, 1,000 ≤ X< 2,000, and 2,000 ≤ X< 20,000), while it had a significant positive correlation with proportions of DP 6 ≤ X< 32 and 32 ≤ X< 62, suggesting that the difference of relative crystallinity among rice from different regions was attributed to starch components and fine structure. Altered relative crystallinity would inevitably change rice RVA parameters, starch solubility, and swelling power, thereby affecting rice cooking and eating quality, which were universally verified by other studies (Cao et al., 2018; Hu et al., 2020).

RVA test was used to simulate the rice cooking process, and it has been widely applied to assess rice cooking and eating quality (Tao et al., 2019; Ma et al., 2021; Li et al., 2022). It is generally considered that rice with excellent eating quality frequently shows an overall trend toward high peak viscosity and breakdown and lower setback (Hori et al., 2016; Balindong et al., 2018). Pasting temperature is a useful indicator to reflect rice cooking quality; rice with a lower pasting temperature requires less energy to complete the gelatinization process than that with a higher pasting temperature. In this study, BD was detected to be negatively correlated with AAC, AM–TS ratio, and ratios of each amylose chain-length section and to be significantly negatively correlated with DP 6 ≤ X< 32 and 32 ≤ X< 62, whereas SB and Cos were both significantly positively correlated with AAC, AM–TS ratio, and proportions of each amylose chain-length section; SB and Cos were significantly negatively correlated with shorter amylopectin chain (DP 6 ≤ X< 32). The above results indicated that lower amylose content and shorter amylopectin chains may both contribute to the softer and sticky texture of YRDCN rice. Pasting temperature was found to be significantly positively correlated with intermediate and longer amylopectin chains and positively correlated with the ratio of longer amylose chain-length sections. Hence, lower pasting temperatures of NECN rice varieties may attributed to their higher amylose contents. Moreover, some NECN rice varieties (JG816, JG515, and JND667) also showed lower pasting temperatures even though they had similar amylose content to most tested Japanese rice varieties. This phenomenon may be explained by their higher proportion of amylose branches ranging from 2,000 to 20,000 DP.

**Figure 2:**
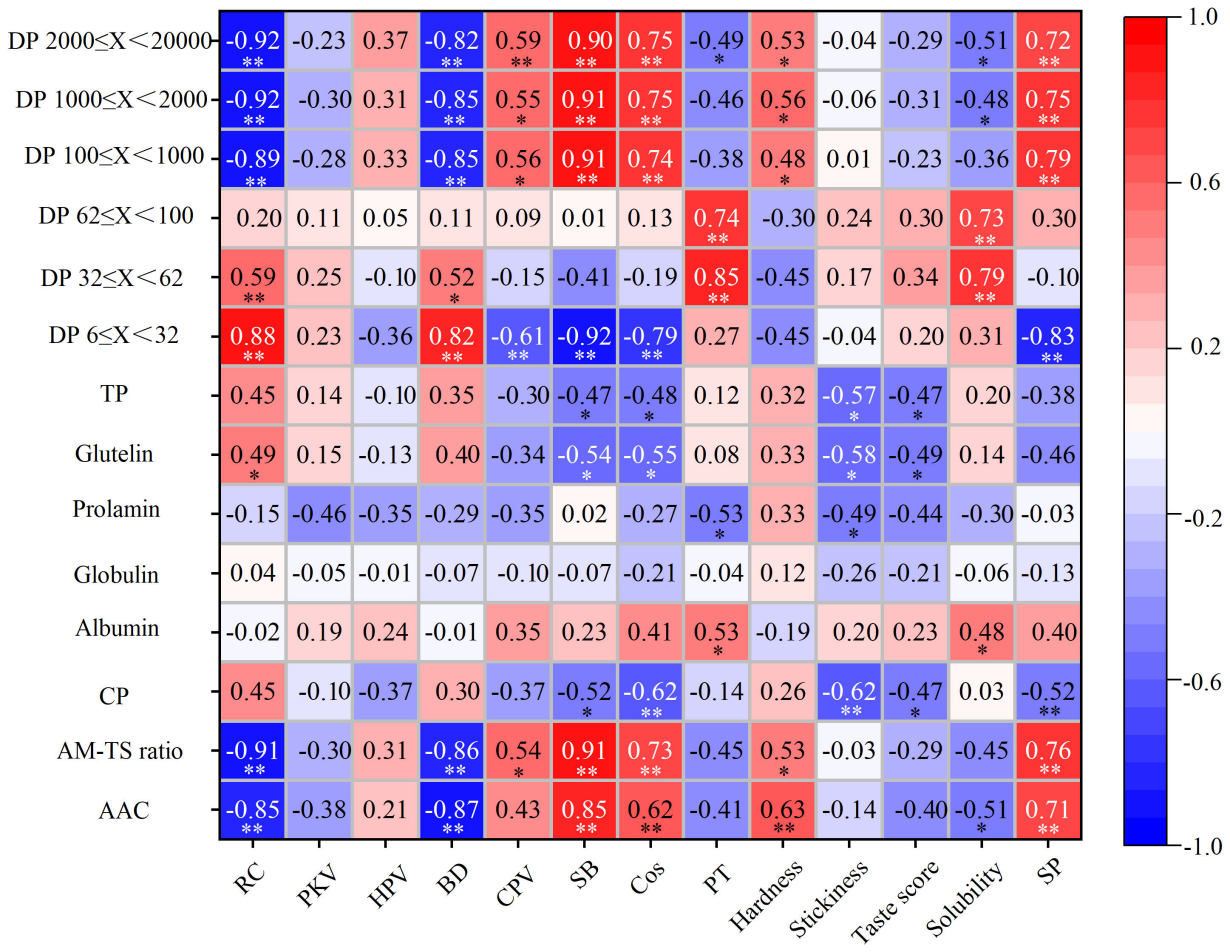
Pearson’s correlation coefficients between rice cooking and eating parameters and grain components and starch fine structure parameters. AAC, apparent amylose content; AM–TS ratio, amylose–total starch ratio; CP, crude protein; TP, total protein, the sum of the four components; RC, relative crystallinity; PKV, peak viscosity; HPV, hot paste viscosity; BD, breakdown; CPV, cool paste viscosity; SB, setback; Cos, consistency; PT, pasting temperature; SP, swelling power. * Correlations are significant at *p*< 0.05. ** Correlations are significant at *p*< 0.01.

We believed that lower solubility of NECN rice was relevant to its higher amylose content, since amylose is more difficult to solubilize in hot water (Mizukami et al, 1999). The correlation analysis of this research verified the significant negative relationship between AC and solubility (**Figure 2**). The average solubility difference between YRDCN and Japanese rice was not significant as amylose content. Indeed, it did not represent that any two varieties from each group were similar to each other based on the solubility value of specific variety comparison. The swelling degree of starch granules depends on the binding ability of starch molecules to water molecules through hydrogen bonds. Correlation analysis proved that lower swelling power for YRDCN rice was closely associated with its lower amylose content and AM–TS ratio, which was consistent with the results described in previous literature (Vandeputte et al., 2003; Wang et al., 2020). Since amylose molecules are mostly located in the amorphous region of starch granules and are exposed more easily to combine with water molecules than amylopectin during the pasting process, rice with higher amylose content usually shows a higher swelling degree. Proportions of amylose DP 100 ≤ X< 1,000, 1,000 ≤ X< 2,000, and 2,000 ≤ X< 20,000 were detected to be positively correlated with swelling power, while the proportion of amylopectin DP 6 ≤ X< 32 was negatively correlated with swelling power. This was probably the affiliated effect of the positive relation between amylose content and swelling power, as this part occupies the majority of rice amylose.

The rice taste analyzer (STA1A) manufactured by SATAK company has been a popular tool for estimating japonica rice eating quality due to its rapidity, repeatability, and objectivity. In this study, each tested variety scored at least 70 points, and their taste values surpassed that of the most common high-quality japonica rice varieties in China according to recent reports (Ma et al., 2021; Zhu et al., 2022), further proving that the tested 12 varieties from two regions of China in this experiment could well represent the current premium varieties. Results of this study and other literature (Ma et al., 2021; Shi et al., 2022) demonstrated the negative impact of protein content on rice taste value. Although YRDCN rice varieties have higher protein contents, this group of rice even generally presents a bit higher taste values than NECN rice. This may be associated with their lower amylose content as presented in correlation analysis (**Figure 2**) because amylose also played an important role in the taste value evaluation standard of rice taste analyzer in addition to protein, which is based on physicochemical measurements using near-infrared reflectance. Evidently, the high taste value of the rice variety was necessarily accompanied by lower hardness and higher stickiness in the assessment system of the rice taste analyzer. A slight hardness mouthfeel did not represent terrible palatability for everyone since rice consumers’ preference for cooked rice texture depends on regions, sociocultural factors, and others (Custodio et al., 2019).

### 4.4 Improvement directions for YRDCN japonica rice quality

Since 2010, semi-glutinous rice has developed rapidly, with a perennial planting scale of over 15 million acres in Jiangsu province, the second-largest japonica rice-producing region in China. In recent years, semi-glutinous rice has been universally preferred by residents in YRDCN. Although semi-glutinous japonica rice had obtained good fame in the YRDCN rice market, its dull endosperm appearance limited its commodity value. The appearance shortage of semi-glutinous japonica rice is expected to be modified by controlling grain moisture or genetic editing as reported in recent literature (Zhang et al., 2019; Zhang et al., 2021b). This study testified to the progress in protein content control for YRDCN rice, but we thought that there still was considerable room for decreasing rice grain protein content. Farmers from YRDCN used to apply larger quantities of nitrogen fertilizer to obtain high yields (Zhao et al., 2012; Guo et al., 2017), and this was probably another core reason for undesirable high grain protein content in addition to higher grain-filling temperature in this region. Admirably, this tendency had been partly inhibited by the extension of rice high-quality cultivation techniques and the price advantage of premium rice to common rice, which limited farmers’ nitrogen application amounts. Relative to conventional urea fertilizing practice with three or four times’ applications in YRDCN, the controlled-release nitrogen application technique was found to be a useful measure for improving rice nitrogen use efficiency while maintaining yield (Xu et al., 2022). We considered that this may also be a useful measure to reduce rice grain protein content due to its lower total nitrogen application quantity, which is required to be verified in future studies. In addition, the eurytopicity of YRDCN rice taste also needed to be respected since a too-soft mouthfeel hardly obtains sufficient recognition from consumers outside YRDCN, especially the residents from indica rice-producing regions. Starch synthesis-related genes determine amylose and amylopectin amounts and their chain-length distribution and thereby affect the functional properties of rice starch. For example, *GBSSI* is encoded by the *waxy* gene in cereals, and it is significantly responsible for the synthesis of linear chains in amylose (Gaur et al., 2023), while *SSIIa* greatly contributes to the synthesis of intermediate single-lamella amylopectin (Wang et al., 2015). Future studies should concern the role of starch synthesis-related genes in rice grain quality difference from different regions in order to provide genetic modification improvement direction for rice grain quality.

## 5 Conclusion

This research investigated japonica rice quality and physicochemical properties from YRDCN, NECN, and Japan. Collectively, each group of japonica rice showed clearly different quality characteristics: japonica rice from Japan presents values of rice components and starch fine structure parameters usually between YRDCN and NECN. NECN japonica rice varieties varied more largely internally when compared with the other two groups. YRDCN rice had a weaker appearance, whereas it did not show inferior cooking and eating quality than NECN and Japanese rice in instrument evaluation systems, which was primarily linked to lower amylose content. Rice amylose content and starch fine structure significantly correlated with RVA parameters, swelling power, and solubility, while protein and glutelin contents had a close relation with taste value parameters; they jointly contributed to the disparity of cooking and eating parameters of japonica rice from three production regions. Limited by the lack of enough experienced panelists, it is regrettable that this research did not evaluate cooked rice sensory properties by artificial tasting.

## Data availability statement

The raw data supporting the conclusions of this article will be made available by the authors, without undue reservation.

## Author contributions

ZD: Data curation, Formal analysis, Funding acquisition, Project administration, Software, Writing – original draft, Writing – review & editing. QY: Data curation, Formal analysis, Project administration, Conceptualization, Writing – review & editing. HaG: Data curation, Project administration, Writing – review & editing. YZ: Data curation, Project administration, Writing – review & editing. QX: Data curation, Project administration, Software, Writing – review & editing. HuG: Funding acquisition, Writing – original draft.
